# Patient Activation and Glycemic Control Among Filipino Americans

**DOI:** 10.1089/heq.2020.0075

**Published:** 2021-04-05

**Authors:** Razel B. Milo, Arlin Ramira, Patricia Calero, Jane M. Georges, Alexa Pérez, Cynthia D. Connelly

**Affiliations:** ^1^ Hahn School of Nursing and Health Science, Beyster Institute for Nursing Research, University of San Diego, San Diego, California, USA.; ^2^ Department of Nursing and Health Occupations, Higher Education Center at Otay Mesa, Southwestern College, San Diego, California, USA.

**Keywords:** PAM-13, diabetes mellitus, Filipino, patient activation, self-management

## Abstract

**Purpose:** Increasing patient activation facilitates self-management of health, improves health outcomes, and lowers health care expenditures. Extant research notes mixed findings in patient activation by race/ethnicity. The purpose of the study was to examine the relationships among patient activation, select patient characteristics, and glycemic control among Filipino Americans.

**Methods:** A cross-sectional study was conducted with a convenience sample of Filipino Americans (*n*=191), with a diagnosis of diabetes mellitus type 1 or type 2, recruited from a southern California adult primary care clinic between December 2017 and March 2018. Patient activation, select characteristics, and hemoglobin A1c (HbA1c) levels were assessed. Bivariate and logistic regression analyses were used to identify correlates of glycemic control. The Strengthening the Reporting of Observational studies in Epidemiology (STROBE) checklist was used to develop the study.

**Results:** Participants with HgbA_1_C≤7.0% reported statistically higher patient activation measure (13 items) (PAM-13) natural log score (mean [*M*]=60.32, standard deviation [SD]=13.50) compared to those with an HgbA_1_C>7.0%, *M*=52.58, SD=10.19, *F*(1)=11.05, *p*<0.001. Multivariate logistic regression using age, low-density lipoprotein, and PAM-13 natural log was statistically reliable distinguishing between A_1_C≤7.0 and A_1_C>7.0, −2 LogLikehood=1183.23, *χ*^2^(3)=15.44, *p*<0.001.

**Conclusions:** Patient activation is an important factor in supporting glycemic control. Findings support interventions to target patient activation. Providers are encouraged to use racial/ethnic-centered engagement strategies in resolving health disparity with racial and ethnic minorities to facilitate patient activation and improve health outcomes in patients with diabetes.

## Introduction

Based on 2018 estimates, in the United States (U.S.) ∼34.1 million adults 18 years of age or older, or 13% of all adults, have diabetes mellitus and 7.3 million adults who met laboratory criteria for diabetes were not aware of or did not report having diabetes (undiagnosed).^1^ The associated health care cost of diabetes in the United States reached $327 billion dollars in 2017.^2^ Prevalence of diagnosed diabetes was highest among Native Americans and Alaska Natives (14.7%), people of Hispanic origin (12.5%), and non-Hispanic blacks (11.7%), followed by non-Hispanic Asians (9.2%) and non-Hispanic whites (7.5%). A recent study identified significant differences in rates of diagnosed and undiagnosed diabetes between subgroups of Asian Americans and Latinos, who collectively represent 23% of the U.S. population and are expected to account for 38% by 2060.^3^ Among non-Hispanic Asians, Asian Indians (12.6%) and Filipinos (10.4%) had the highest prevalence of diagnosed diabetes, followed by other Asian groups (9.9%) and Chinese (5.6%).^1^ Among adults, prevalence varied significantly by education level, which is an indicator of socioeconomic status.^1^

Ann Albright, director of the Centers for Disease Control and Prevention (CDC)'s division of diabetes translation, argues these findings establish a baseline for future estimates of diabetes prevalence among Hispanics and non-Hispanic Asian subgroups and highlight differences in diabetes burden among these subgroups. Further research is needed among these subgroups to strengthen diabetes detection and provide care with tailored, effective early prevention and treatment strategies.^3^

Diabetes mellitus is a chronic disease requiring patient involvement in his or her care. Diabetes self-management provides the basis for patients to control complications resulting from unmanaged or poorly managed diabetes. Welch, Zagarins, Feinberg, and Garb found diabetes self-management improves hemoglobin A1c (HbA1c) level between −0.6% to −0.7% in patients with type 2 diabetes mellitus (T2DM).^4^ Extant research shows self-management education for diabetes improves health outcomes.^5,6,7^ Partnership and clear discussion between health care providers and patients are essential to achieve healthy glycemic control.^8^ Consequently, the American Diabetes Association (ADA) position statement asserts continuing patient self-management education and partnership are crucial to prevent diabetes complications.^2^

The concept of *patient activation* is defined as the individual's understanding of one's role in managing health care needs.^9^ It is associated with positive health outcomes, healthy lifestyle and behavior patterns,^10^ and cost-effective utilization of health services.^11,12^ Patient activation requires a multidisciplinary approach to empower patients to embrace self-management.^13,14^ The relationship among the patient, provider, and health care delivery environment shapes both patient engagement process and behavior.^15,16^ Ideally, engagement creates patient-centered care that is respectful of and responsive to individual patient preferences with the patient as an integral member of the care team, and allows for patients to receive information about their health and contribute information that informs their care.^16,17^ Patient-centered care is designed to increase levels of trust and more confidence with providers.^18^ Increasing patient participation and encouraging patient engagement are designed for higher patient and family satisfaction, greater empowerment, better health outcomes, and lower health care expenditures.^15,19^

The patient activation measure (PAM) is a standardized instrument designed to capture whether the patient is ready, able, and interested to engage in self-management of his or her disease.^20^ Previous studies have examined patient activation and self-management of a number of diseases, including diabetes. Do et al. found patient activation was associated with engagement in heart failure self-management.^21^ In a sample of individuals with diabetes and chronic kidney disease, Zimbudzi et al. found a high level of patient activation was positively associated with a higher overall level of self-care.^22^ Hendriks and Rademakers investigated patient activation and its relationships with patient characteristics and health-related outcomes to provide further insight into the gains expected if patients are more involved in their health care.^23^

Participants (*n*=1845) completed a survey that included the PAM, patient characteristics, clinical values, lifestyle and self-management behaviors, knowledge about diabetes, and health care utilization. Findings indicated those with low patient activation level less often reported having knowledge about diabetes and their values on clinical indicators.^23^ Bilello et al. found patients with diabetes, who were in good health, had higher PAM scores.^24^ Woodard, Landrum, Amspoker, Ramsey, and Naik found patient activation was predictive of better HbA1c level independent of other covariates.^25^ Hibbard et al. reported a tailored diabetes management intervention increased the activation score.^26^

Bos-Touwen et al. sought to increase their understanding of characteristics associated with patient activation for self-management and to evaluate whether these are disease-transcending.^27^ A cross-sectional survey study was conducted in primary and secondary care in patients with T2DM, chronic obstructive pulmonary disease (COPD), chronic heart failure (CHF), and chronic renal disease (CRD). They sampled 1154 patients, with 53% response rate: 422 T2DM patients, 290 COPD patients, 223 CHF patients, and 219 CRD patients. Multiple linear regression analysis revealed nine explanatory determinants of activation for self-management (age, body mass index, educational level, financial distress, physical health status, depression, illness perception, social support, and underlying disease), which explained 16.3% of variance in the sample.

These findings provide an argument for supporting clinicians and researchers in identifying subpopulations of chronic disease patients less likely to be engaged in self-management.^27^ Mayberry et al. found a high level of activation in a primary care clinic of highly educated adults, despite their not attending a formal program for diabetes self-management.^28^ Increased research efforts are needed to explain the significant factors that contribute to the complex nature of patient activation for self-management.

Pender's Health Promotion Model defines health as a dynamic positive state aimed at increasing patient's level of well-being, as well as ascribing multidimensional nature of person as he or she relates with his or her environment.^29^ The model's emphasis is on three areas: individual characteristics and experiences, behavior-specific cognition and affect, and behavioral outcomes. The overarching objective of the Health Promotion Model is healthy behavior as the desired behavioral outcome. For example, prior research shows a significant association between lower HbA1c level and self-management behaviors.^30^ A randomized control study conducted by Shi et al. found improved glycemic control self-efficacy among Chinese patients with T2DM.^30^

Consumer and patient activation theory postulates supportive environments, which promote self-management of one's health, are likely to have higher activated consumers than a nonsupportive environment.^31^ Confidence in one's own ability to manage the role of proactive self-manager is a necessary step to become activated. The final assumption of activation theory is the ability to manage one's own health becomes an individual self-concept.

Previous research has noted differences in PAM scores among people from different racial/ethnic backgrounds. Cunningham, Hibbard, and Gibbons examined a sample of blacks, whites, and Latinos on how confident, skillful, and knowledgeable they were about taking an active role in improving their health and health care.^32^ Findings indicated patient activation among blacks and Latinos was low, compared to whites. Fewer Latinos (24.8%) were at the highest level of patient activation, compared to 39.5% of blacks and 45.3% of whites. Among Latino immigrants, low acculturation and lack of familiarity with the U.S. health care system may have contributed to low activation. Patients from minority backgrounds tend to have lower PAM scores, which may be associated with less equitable patient-physician collaboration compared to whites.

The concept of improving one's PAM score is an important approach to change patient behavior and attitude toward self-management.^33^ Notably, patients with an active role in self-management of their disease had higher PAM scores and higher levels of activation compared to patients assuming the passive and collaborative roles.^34^ Woodard et al. found improved HbA1c and self-management among veterans who had better functional health literacy and patient activation.^25^

Management of patients with chronic disease, for example, diabetes self- management, is the foundation in avoiding complications, consequently improving health care and clinical outcomes, and reduced health care cost.^12^ Research conducted by Yang et al. indicated increased knowledge was associated with normal HbA1c in Chinese patients.^35^ Kaplan, Billimek, Sorkin, Ngo-Metzger, and Greenfield research with Mexican Americans and Vietnamese Americans found disparity in care and its relationship to poor glycemic control.^36^ There is, however, a scarcity of research on patient activation among ethnic minority populations compared to whites and information on patient activation for Filipinos is lacking.^33,34^

The Filipino immigrant community in the United States has increased from 1.1% in 1960 to 4.5% in 2013.^37^ The Filipino American population in the United States is the third largest foreign-born population from Asia, after India and China. Filipino immigrants are more likely to have a strong command of the English language, be college educated, have higher income, and have lower poverty rates, and less likely to be uninsured compared to other immigrants.^37^ This supports the need for further investigation of the patient activation concept among Filipinos.

The dearth of knowledge about their activation practices and the new knowledge gained from this research will provide preliminary information for understanding and future development of early prevention and intervention strategies for resolving health disparities among ethnic minorities.^38^ The purpose of this study was to examine the relationship among patient activation, select demographic variables (i.e., age, gender, and level of education), low-density lipoprotein (LDL) level, and self-management of HbA1c among Filipinos with type 1 diabetes mellitus and T2DM.

## Methods

A prospective cross-sectional design was used for this study.

### Setting and sample

A convenience sample was recruited and enrolled from an adult primary care clinic located in southern California. Inclusion criteria were as follows: Filipino descent, 18-years or older, diagnosed with diabetes, currently seeing a primary care provider at the participating primary care clinic, available laboratory results for HbA1c and LDL levels, able to speak or comprehend instructions in English or Tagalog, and willingness to participate in all aspects of the research study. The study was developed using the Strengthening the Reporting of Observational studies in Epidemiology (STROBE) checklist for cross-sectional studies as provided in the Supplementary Material.

### Procedures and measures

Participants were recruited through flyers posted at the participating primary care clinic. Interested participants, who met inclusion criteria, were provided a packet containing an informed consent form, a demographic questionnaire, and PAM-13 to complete. The principal investigator was onsite to present the research project, and facilitate obtaining the informed consent and data collection.

#### Patient activation measure

Patient activation was measured using the PAM-13 developed by Hibbard et al.^19^ Permission to use this instrument was obtained through Insignia Health.^20^ The PAM-13 measures a latent construct reflecting individual's perception of one's ability to manage self-care needs. Research purports patient activation is a significant predictor of health care behavior, clinical indicators, and health care cost.^39^ The measure is a self-administered questionnaire, with response options ranging from 1 (strongly disagree) to 4 (strongly agree), and an additional 0 (not applicable) option. An activation score is calculated by dividing the raw scores by the number of items answered and multiplied by 13.

Scores range from 0 (no activation) to 100 (high activation) and higher scores indicate increased patient activation and positive associations with adaptive healthy behaviors.^40,41^ Raw scores can be converted into activation levels ranging from 0 (no activation) to 4 (high activation). Patients with lower activation scores may lack the belief and knowledge required to manage their chronic illness, while patients with high activation scores may have complete confidence and knowledge to manage their condition (Table 1).^19^ The PAM-13 is a reliable and well-validated measure for assessing patient activation in healthy patients and chronically ill patients, with a Cronbach's alpha of 0.88, evidencing good internal consistency.^22,40,42,43^

**Table 1.** Patient Activation Measure (13 Items) Scoring System and Levels of Progressive Activation

Level	Score	Definition	Behaviors
Level 1	≤47.0	Not believing activation is important	Disengaged and overwhelmed
			Passive
			Lacks confidence
			Knowledge is low
			Weak goal orientation
			Poor adherence
Level 2	47.1–55.1	Lack of knowledge and confidence to take action	Becoming aware, but still struggling
			Some knowledge, large gaps remain
			Can set simple goals
Level 3	55.2–67.0	Beginning to take action	Taking action and gaining control
			Have the key facts
			Building self-management skills
			Goal oriented
			Strive for best practices
Level 4	≥67.1	Taking action	Maintaining behaviors and pushing further
			Adopted new behaviors, but may struggle during times of stress or change
			Maintaining a healthy lifestyle is key focus

References: Skolasky et al.^43^; Moljord et al.^51^

#### Clinical indicators of glycemic control

Patients' biological markers (e.g., HbA1c and LDL levels) were collected by a laboratory service contracted by the primary care physician; results were entered in patients' electronic health record. Participants entered the results (within the last 6 months) of their last HbA1c and LDL levels in the self-administered demographic questionnaire; with results verified by the principal investigator using patients' electronic health record.

The acceptable normal range for HbA1c level is <7% over the last 3–6 months.^44,45^ HbA1c level was measured as a continuous variable and categorized based on a cutoff score of 7% (≤7=successful glycemic control and >7=poor glycemic control). The optimal level of LDL suggested by the National Institute of Health, National Heart, Lung, and Blood Institute is <130 mg/dL every 5 years for young adults, and every 1–2 years for males (ages 45–65 years) and females (ages 55–65 years).^46^

#### Demographic questionnaire

A questionnaire was created for this study using Pender's Health Promotion Model,^28^ and the review of literature. Items included age, gender, highest level of education, and preferred spoken and written language.

### Data analysis

The sample size was calculated using Power G*3.1.9.2 with six independent variables/covariates to be tested in a multiple linear regression model. This sample size was considered sufficient to detect a moderate effect size (*f*^2^=0.15) using a two-tailed significance test with a power of 0.80 and a significance level of 0.05.^47^ To account for attrition, a total of 191 participants were recruited and enrolled. Descriptive statistics were calculated for all analysis variables and data were examined for normality, missing values, and outliers.

Reliability of PAM-13 activation score was evaluated by Cronbach's alpha coefficient, and compared to the coefficients described in the literature. Bivariate associations were examined with chi-squared analysis for categorical variables, and one-way analysis of variance (ANOVA) for continuous variables. Variables significant at *p*<0.05 in the bivariate analysis and those significant in the review of the literature were considered for entry into a logistic regression model to identify factors that increase the likelihood of successful diabetic self-management (HbA1c <7). All statistical analyses were performed with IBM SPSS software version 23.0 (IMB Corp., Armonk, NY).

### Ethical considerations

Before study initiation, in accordance with the U.S. Code of Federal Regulations on the Protection of Human Subjects (45 CFR 46 and 21 CFR 50 and 56), all study procedures were reviewed and approved by the appropriate administrative and university Institutional Review Boards. All participants gave written informed consent before partaking in any study activity.

## Results

### Demographic characteristics

One hundred ninety-one individuals were recruited and enrolled. Females represented slightly more than two-thirds (65.4%, *n*=125) of the sample. Mean age of participants was 67.95 (standard deviation [SD]=14.17, range 23–102 years). More than half (54.5%, *n*=104) reported having college or higher than college education; only one participant indicated having a grade school education. Surprisingly, 93.7% (*n*=179) selected Tagalog as their preferred spoken or written language, with only 6.3% (*n*=12) completing the survey in English. Patient activation scores ranged from 28.21 to 217 (mean [*M*]=58.8, SD=13.21). Patient activation levels were as follows: 13.6% patients (*n*=26) in level 1, 30.4% (*n*=58) in level 2, 39.3% (*n*=75) in level 3, and 16.8% (*n*=32) in level 4. LDL values ranged from 33 mg/dL to 217 mg/dL, and 16% (*n*=30) had an LDL level >130 mg/dL. HbA1c values ranged from 5.10 to 8.0, and 78.5% of patients (*n*=150) had HbA1c values ≤7%.

### Glycemic control

Demographic characteristics overall and by HbA1c group are presented in Table 2. Chi-square test of independence and one-way ANOVA were conducted to determine if patient activation was significantly different for groups with different HbA1c levels and scores. Participants were classified into two groups: successful glycemic control (HbA1c ≤7%) and poor glycemic control (HbA1c >7%). Chi-square test results showed that patient activation levels were significantly associated with glycemic control, *χ*^2^(3)=18.66, *p*<0.001. For this sample, 20% (*n*=30) of patients had successful glycemic control (HbA1c ≤7) compared with only 4.9% (*n*=2) who had poor glycemic control (HbA1c >7).

**Table 2.** Demographic and Clinical Characteristics of Study Population by Hemoglobin A1c Group (*n*=191)

Characteristic	Total		Successful GC HbA1c ≤7		Poor GC HbA1c >7		Χ^2^	p
	n	%	n	%	n	%n		
Gender							0.187	0.665
Male	66	34.6	53	35.3	13	31.7		
Female	125	65.4	97	64.7	28	68.3		
Education level							0.013	0.909
Grade, high school	87	45.5	68	45.3	19	46.3		
College, higher	104	54.5	82	54.7	22	53.7		
Preferred language							1.31	0.252
English	12	6.3	11	7.3	1	2.4		
Tagalog	179	93.7	139	92.7	40	97.6		
PAM-13 levels							18.66	<0.001
Level 1, ≤47.0^a^	26	13.6	16	10.7	10	24.4		
Level 2, 47.1–55.1^b^	58	30.4	38	25.3	20	48.8		
Level 3, 55.2–67.0^c^	75	39.3	66	44.0	9	22.0		
Level 4, ≥67.1^d^	32	16.8	30	20.0	2	4.9		
	***M***	**SD**	***M***	**SD**	***M***	**SD**	***F***	***p***
Age, years	67.95	14.17	67.66	14.35	69.02	13.66	0.290	0.588
LDL, mg/dL	100.12	33.10	99.53	31.10	102.26	40.05	0.218	0.641
PAM-13 score	58.71	13.21	60.32	13.50	52.78	10.19	11.06	0.001

^a^ Level 1: not believing activation is important.

^b^ Level 2: lack of knowledge and confidence to take action.

^c^ Level 3: beginning to take action.

^d^ Level 4: taking action.

GC, glycemic control; HbA1c, hemoglobin A1c level; LDL, low-density lipoprotein; *M*, mean; PAM-13, patient activation measure (13-items); SD, standard deviation.

Gender, level of education, and language preference were not significantly associated with HbA1c groups. Similarly, one-way ANOVA test results indicated that patient activation scores were significantly higher for those with better glycemic control (*M*=60.32, SD=13.50) compared to those with poor glycemic control, *M*=52.58, SD=10.19, *F*(1)=11.05, *p*<0.001. Not surprisingly, patient activation measured as a continuous score and activation levels were highly correlated, *r*=0.86, *p*=0.001. Two separate regression models were generated.

A binomial logistic regression was conducted to ascertain the effects of age, LDL level, and patient activation score on the likelihood that participants achieve successful glycemic control. A test of the overall model against a constant-only model was statistically significant, *χ*^2^(3)=15.44, *p*<0.001. The model correctly classified 78.5% of the cases. Regression coefficients are presented in Table 3. Of the three predictor variables, only patient activation was statistically significant, predicting successful diabetes self-management. The odds ratio indicated that low activation scores were associated with poorer glycemic control (higher HbA1c levels).

**Table 3.** Summary of Logistic Regression Analysis Predicting Successful Diabetes Self-Management Hemoglobin A1c Using Patient Activation Score (*n*=191)

Variable	B	SE	OR	95% CI	Wald statistic	p
Age	−0.02	0.02	0.98	0.95–1.01	2.07	0.150
LDL	>0.01	0.01	1.00	0.99–1.01	0.02	0.895
PAM-13 score	−0.08	0.02	0.92	0.88–0.97	10.71	0.001
*χ*^2^(3)=15.44, *p*<0.001						
−2 Log likelihood=183.23. Nagelkerke *R*^2^=0.120						

CI, confidence interval for OR; OR, odds ratio; SE, standard error.

A second binomial logistic regression was conducted to determine the effects of age, LDL level, and patient *activation levels* on the likelihood that participants achieve successful glycemic control. A test of the overall model against a constant-only model was statistically significant, *χ*^2^(3)=19.98, *p*<0.001. The model correctly classified 79.6% of the cases. Regression coefficients are presented in Table 4. Once again, of the three predictor variables, only patient activation was statistically significant, predicting successful diabetes self-management. The odds ratio indicated once again patients with low levels of activation had poorer glycemic control (higher HbA1c levels).

**Table 4.** Summary of Logistic Regression Analysis Predicting Successful Diabetes Self-Management Hemoglobin A1c Using Patient Activation Levels (*n*=191)

Variable	B	SE	OR	95% CI	Wald statistic	p
Age	−0.03	0.02	0.98	0.94–1.00	2.84	0.092
LDL	<0.01	0.01	1.00	0.99–1.01	0.01	0.925
PAM-13 level 1	2.98	0.92	17.72	2.91–107.7	9.78	0.002
PAM-13 level 2	2.56	0.84	13.01	2.53–66.84	9.44	0.002
PAM-13 level 3	0.94	0.83	2.56	0.51–12.87	1.30	0.255
*χ*^2^(5)=22.82, *p*<0.001						
−2 Log likelihood=175.84. Nagelkerke *R*^2^=0.174%						

PAM-13 level 4 is the reference category.

## Discussion

Patient activation is associated with positive health outcomes, healthy lifestyle and behavior patterns,^10^ and cost-effective utilization of health services.^11,48^ Results of this study provide needed initial exploration of patient activation in a Filipino American sample. The research examined the relationship among patient activation, select demographic variables, LDL, and self-management of HbA1c among Filipino Americans with diabetes. The relationship between patient activation in this Filipino American sample was associated with self-management of HbA1c. Notably, patient activation was predictive of better HbA1c levels independent of other covariates, supporting the work of Hendriks and Rademakers, Bilello et al., and Woodard et al., who found patient activation was predictive of better HbA1c levels.^23,24,25^

Study findings must be placed in the context of study limitations. The nature of the cross-sectional design makes it impossible to discern causality. Although there was adequate sampling of participants, a convenience sampling was used. Notably, in our study, education, which is identified as a significant predictor of glycemic control in other studies and is included in the ADA recommendations, was not statistically significant, *χ*^2^=0.013, *p*=0.909. It appears education is not significant because of the sample makeup. The participants (*n*=179) who responded in Tagalog were fairly evenly distributed for education level, with 87 reporting high school or less and 92 reporting college in comparison to the 12 who responded in English, and all reported college, *χ*^2^(1)=10.7, *p*=0.001. It can be speculated that the lack of disease knowledge and management could affect glycemic control.

Of note, participants were recruited during primary care visits with a health care provider and had similar access to health care resources. Further research with a sample with limited or no access to health care services is warranted. Other factors missing from our data were marital status, family history of diabetes, underlying disease knowledge, and stigma; all indicators influence self-management of diabetes. Future research should examine the relationships among knowledge of disease, stigma, and specific patient characteristics, including family diabetes history, marital status, and HbA1c levels. Despite these limitations, findings provide valuable information and suggest potential new directions for research to reduce racial and ethnic health disparities through culturally tailored diabetes self-management.

### Health equity implications

Careful attention should be considered in recognizing subtle differences between empowerment and personal responsibility in diabetes self-management, in contrast to blaming individuals for causing their own health problems. Health care professionals need to consider how stigma can cause distress, which leads to poor self-management of the disease. Further research is needed to explore the social experiences of participants with diabetes compared to those without the disease. Understanding the role of culture and managing diabetes in Filipino Americans requires cultural flexibility on the part of medical providers to build rapport and find culturally acceptable middle ground to improve engagement.

Acknowledging cultural beliefs and the role of family members as a resource may facilitate diabetes self-management. The study findings support ADA's clinical recommendations of a holistic approach when caring for patients with diabetes. Clinicians must consider racial and ethnic background, culture, education, values, comorbidities, and co-existing diseases when interpreting guidelines and managing treatment plans.^2^ Further research on patient activation and self-management of HbA1c among Filipino Americans will facilitate the development of early prevention intervention strategies to improve existing programs.

Study findings supporting patient activation in improving diabetic self-management among Filipino Americans are relevant to health equity. In this study, patient activation and HbA1c self-management are important factors in supporting glycemic control in Filipino Americans. Patients who reported confidence in self-management had higher PAM scores. A racial/ethnic-centered mediation on patient activation could lead to better health outcomes in patients with diabetes.

## Conclusion

Diabetes mellitus is a chronic disease commonly associated with long-term complications, which can be prevented with improvement in self-management. This study supports the contribution of patient activation in the reduction of patients' HbA1c. ADA recommendations include an individualized approach to diabetes management based on the patients' racial/ethnic background, culture, education, values, comorbidities, and co-existing diseases.^2^ In addition, there is limited research examining PAMs among patients with diabetes in a diverse population. Consequently, there are limited data on the relationship between patient activation and diabetes management in a diverse population.

Health care clinicians should recognize the impact of patient activation in diabetes self-management. Approaches to improve patients' self-management should include the use of available technology to encourage patient compliance. Future research with a focus on the use of telehealth, text messaging, or voice messaging to improve patient activation in self-management is warranted.^49,50^ Efforts emphasizing culturally sensitive interventions to encourage patient engagement are needed to enhance the quality of life among patients with diabetes.

## Supplementary Material

Supplemental data

## Supplemental Material

Supplementary STROBE checklist for cross-sectional studies

## Abbreviations Used

ADA: American Diabetes Association
ANOVA: analysis of variance
CDC: Centers for Disease Control and Prevention
CFR: Code of Federal Regulation
CHF: chronic heart failure
CI: confidence interval
COPD: chronic obstructive pulmonary disease
CRD: chronic renal disease
GC: glycemic control
HbA1c: hemoglobin A1c level
LDL: low-density lipoprotein
*M*: mean
OR: odds ratio
PAM-13: patient activation measure (13-items)
SD: standard deviation
SE: standard error
STROBE: Strengthening the Reporting of Observational studies in Epidemiology
T2DM: type 2 diabetes mellitus
